# ERRATUM: Breatfeeding and consumption of sweetened foods

**DOI:** 10.1590/1984-0462/;2018;36;4;00019erratum

**Published:** 2019-04-01

In the manuscript “Breatfeeding and consumption of sweetened foods”, DOI: 10.1590/1984-0462/;2018;36;4;00019, published in the Rev Paul Pediatr. 2018;36(4):524-525, page 524:

Where it reads:

Lúcia Campos Pellanda^a,^*

^a^Instituto de Cardiologia, Fundação Universitária de Cardiologia, Porto Alegre, RS, Brazil.

It should read:

Maíra Ribas Goulart^a^, Lúcia Campos Pellanda^a,^*

^a^Instituto de Cardiologia, Fundação Universitária de Cardiologia, Universidade Federal de Ciências da Saúde de Porto Alegre, Porto Alegre, RS, Brazil.

In the manuscript “Risk behavior for bulimia among adolescents”, DOI: 10.1590/1984-0462/;2019;37;2;00008, published in the Rev Paul Pediatr. In press 2019. Epub Feb 25, 2019:

Where it reads:

Amanda Silva Aragãoc

It should read:

Amanda Silva Aragão

